# Supplementary material for: Use and Acceptance of Drinking Fountains: A Pilot Study in Two Secondary Schools in Dortmund, Germany
Source: Children (Basel). 2023 Apr 29;10(5):817. doi: 10.3390/children10050817 (PMC10217575; doi:10.3390/children10050817)
Supplement: Supplementary file 1 [file children-10-00817-s001.zip › children-2347300-supplementary.pdf]

Photo S1. Drinking fountain Hauptschule pictures

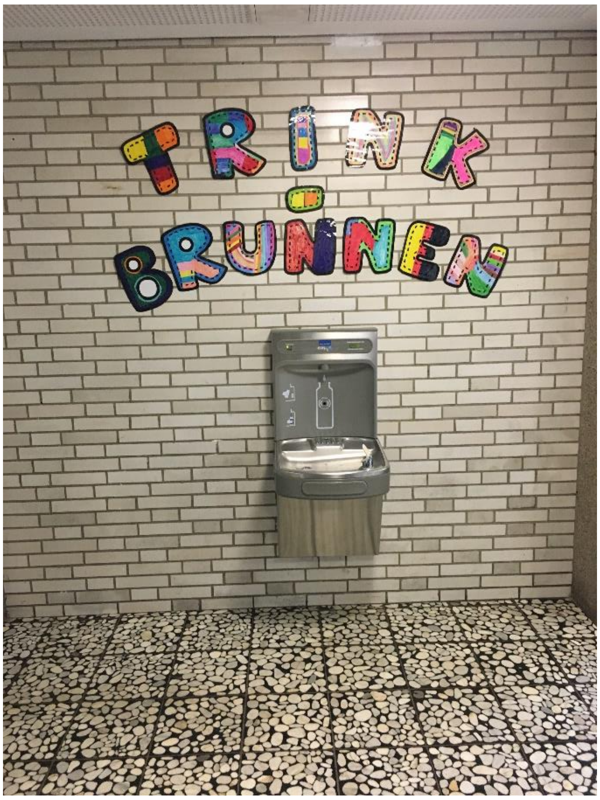

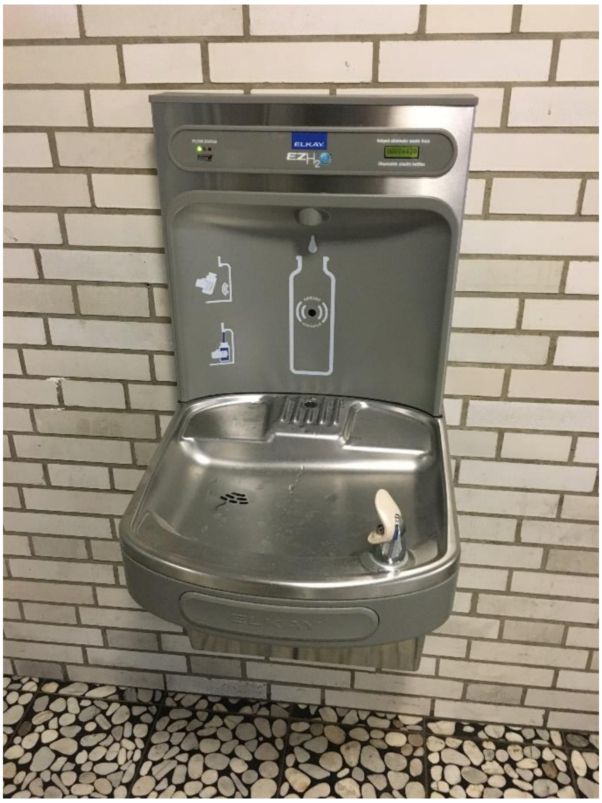

Photo S2. Drinking fountain Gymnasium pictures

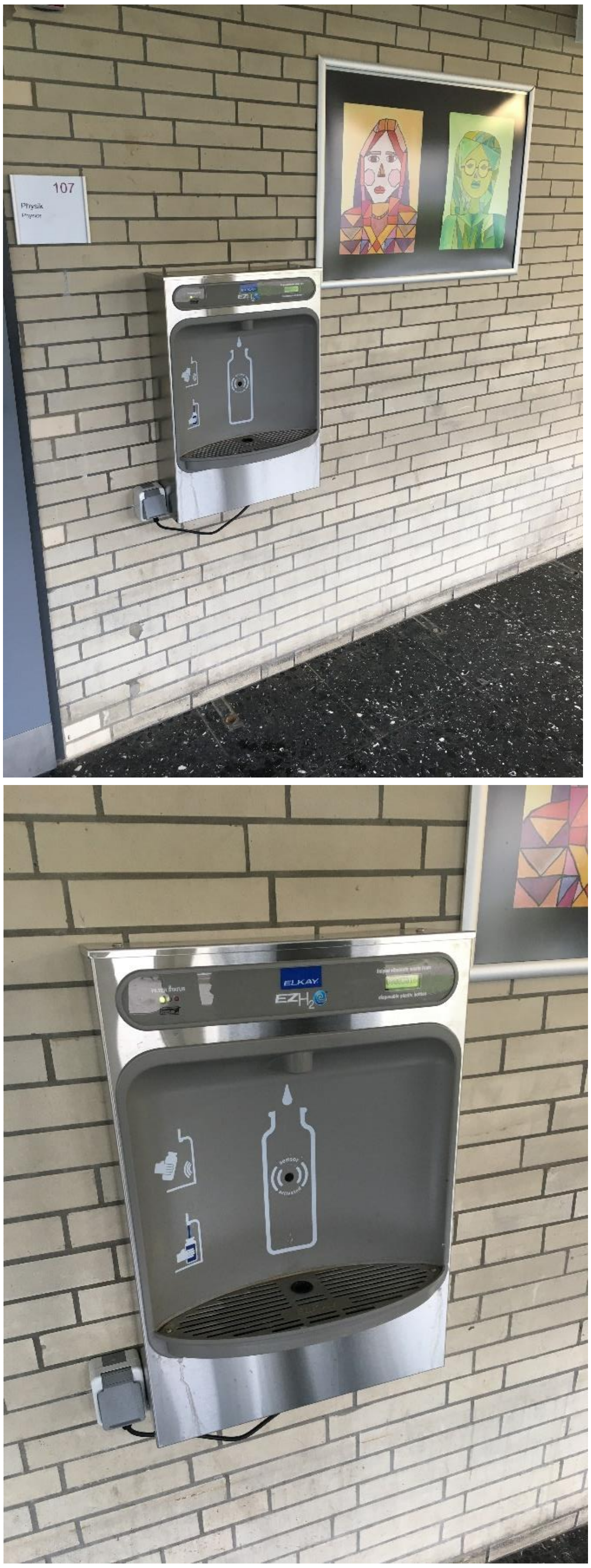

Questionnaire S1. Student questionnaire containing 26 closed and two open items (German and English)

*Fragebogen für Schüler*innen*

Nutzung und Akzeptanz von Trinkbrunnen an Dortmunder Schulen

Liebe Schülerin, lieber Schüler,
toll, dass Du dich bereit erklärt hast, uns einige Fragen zum Trinkbrunnenprojekt an eurer Schule zu beantworten. Wir wollen mit dieser Studie herausfinden, wie oft der Trinkbrunnen an Eurer Schule genutzt wird, und ob es Dinge gibt, die man besser machen könnte. Außerdem interessiert uns, wie wichtig Dir gesunde Getränke sind.
Niemand kann im Nachhinein herausfinden, zum wem welcher Fragebogen gehört.

Zunächst möchten wir gerne einige generelle Dinge über Dich wissen

1. Ich bin ein…

   ◎[1] Mädchen [2] Junge ◎[3] Divers ◎[9] Möchte ich nicht beantworten

2. Wie alt bist du?

   Ich bin __________ Jahre alt ◎[9] Möchte ich nicht beantworten

3. Auf was für eine Schule gehst du?

   ◎[1] Hauptschule ◎[2] Gymnasium ◎[9] Möchte ich nicht beantworten

4. In welche Klasse / Jahrgangstufe gehst du?

   ◎[1]6
   ◎[2]8
   ◎[3]10
   ◎[9] Möchte ich nicht beantworten

Nun interessiert uns, welche Getränke Du während der Schulzeit trinkst

5. Verwendest du den Trinkbrunnen in der Schule?

   ◎[1] Ja ◎ [2] Nein ◎[9] Möchte ich nicht beantworten

6. Wenn ja, wie oft hast Du in der letzten Woche den Trinkbrunnen genutzt?

◎[1] Mehr als 3-mal pro Tag
◎[2] 1 – 2-mal am Tag
◎[3] Einmal am Tag
◎[4] 1-2-mal die Woche
◎[5] Seltener als einmal die Woche
◎[9] Möchte ich nicht beantworten

______

7. Gab es in Deiner Grundschule einen Trinkbrunnen?

   ◎[1] Ja ◎[2] Nein ◎[9] Möchte ich nicht beantworten

______

8. Bringst du normalerweise Getränke von zu Hause mit?

   ◎[1] Ja ◎[2] Nein ◎[9] Möchte ich nicht beantworten

______

9. Wenn ja, welche Getränke bringst du normalerweise von zu Hause mit? (Hier darfst du gerne mehr als ein Kreuz machen)
   ◎[1] Wasser
   ◎[2] Saftschorlen
   ◎[3] Limonaden
   ◎[4] Koffeinhaltige Limonaden
   ◎[5] Energy – Drinks
   ◎[6] Kakao
   ◎[7] Andere ________________
   ◎[9] Möchte ich nicht beantworten

______

10. Bringst du seltener Getränke von zu Hause mit, seitdem es den Trinkbrunnen an deiner Schule gibt?

    ◎[1] Ja ◎[2] Nein ◎[9] Möchte ich nicht beantworten

______

11. Kaufst du während der Schulzeit normalerweise Getränke? (zum Beispiel im Schulkiosk oder Supermarkt?)

    ◎[1] Ja ◎[2] Nein ◎[9] Möchte ich nicht beantworten

______

12. Wenn ja, welche Getränke kaufst Du?
    ◎[1] Wasser
    ◎[2] Saftschorlen
    ◎[3] Limonaden
    ◎[4] Koffeinhaltige Limonaden
    ◎[5] Energy – Drinks
    ◎[6] Kakao
    ◎[7] Andere ________________
    ◎[9] Möchte ich nicht beantworten

Außerdem möchten wir gerne noch Deine Meinung zu folgenden Aussagen wissen:

		Stimmt voll und ganz[1]	Stimmt teilweise[2]	Stimmt eher nicht[3]	Stimmt gar nicht[4]	Möchte ich nicht beantworten[9]
13.	Trinkbrunnen an unserer Schule sind eine gute Idee	◎	◎	◎	◎	◎
14.	Andere Schulen sollten auch Trinkbrunnen bekommen	◎	◎	◎	◎	◎
15.	Ich trinke mehr Wasser, seitdem es den Trinkbrunnen gibt	◎	◎	◎	◎	◎
16.	Andere Kinder/Jugendliche aus meiner Klasse/Schule nutzen den Trinkbrunnen regelmäßig.	◎	◎	◎	◎	◎
17.	Wenn ich mir ein Getränk auswähle, achte ich darauf, dass es gesund ist	◎	◎	◎	◎	◎

18. Wen fragst Du am ehesten, wenn du Fragen zu gesunden Getränken hast?
    - ◎[1] Meine Eltern
    - ◎[2] Meine Lehrer
    - ◎[3] Meine Freunde
    - ◎[4] Ich suche mir die Informationen im Internet
    - ◎[9] Möchte ich nicht beantworten

		Stimmt voll und ganz[1]	Stimmt teilweise[2]	Stimmt eher nicht[3]	Stimmt gar nicht[4]	Möchte ich nicht beantworten[9]
19.	Ich kann gesunde von ungesunden Getränken unterscheiden	◎	◎	◎	◎	◎
20.	Gesunde Getränke wurden im Unterricht behandelt	◎	◎	◎	◎	◎

Für wie gesund hältst Du die nachstehenden Getränke?
(kreuze immer nur eine Möglichkeit an)

		Gesund[1]	Eher gesund[2]	Eher nicht gesund[3]	ungesund[4]
21.	Wasser	◎	◎	◎	◎
22.	Saftschorle	◎	◎	◎	◎
23.	Limonaden	◎	◎	◎	◎

24.	Koffeinhaltige Limonaden	◎	◎	◎	◎
25.	Energy-Drinks	◎	◎	◎	◎
26.	Kakao	◎	◎	◎	◎

Freitextfragen

27. Gibt es etwas, das Du an den Trinkbrunnen gerne ändern würdest?
(Zum Beispiel wie sie aussehen oder wie sie funktionieren)

____

____

28. Falls Du uns noch etwas Anderes zum Thema Trinkbrunnen mitteilen möchtest, hast Du hier noch Platz:

____

____

VIELEN DANK FÜR DEINE TEILNAHME!

Questionnaire for students

Use and acceptance of drinking fountains at Dortmund schools

Dear student,
Great that you agreed to answer some questions about the drinking fountain project at your school. With this study, we want to find out how often the drinking fountain is used at your school and whether there are things that could be done better. We are also interested in how important healthy drinks are to you.
No one can find out afterwards which questionnaire belongs to whom.

First, we would like to know a few general things about you

1. I am a...

   1 Girl 2 Boy ◎3 Divers ◎9 Do not want to answer

   ______

2. How old are you?

   I am __________ years old ◎9 I do not want to answer this question

   ______

3. What school do you go to?

   1 Hauptschule ◎2 Gymnasium ◎9 I do not wish to answer this question.

   _____

4. Which class / grade are you in?

   16
   28
   310
   9 Do not wish to answer

   ______

Now we are interested in what drinks you drink during school hours

5. Do you use the drinking fountain at school?

   1 Yes ◎2 No ◎9 Would not like to answer

   ______

6. If so, how often have you used the drinking fountain in the last week?

¹ More than 3 times a day
² 1 - 2 times a day
³ Once a day
⁴ 1-2 times a week
⁵ Less often than once a week
⁹ I don't want to answer that

7. Was there a drinking fountain at your primary school?

   ¹ Yes ◎² No ◎⁹ Would not like to answer

8. Do you usually bring drinks from home?

   ¹ Yes ◎² No ◎⁹ Would not like to answer

9. If yes, what drinks do you usually bring from home? (You are welcome to put more than one cross here)
   ¹ Water
   ² Juice spritzers
   ³ Lemonades
   ⁴ Caffeinated soft drinks
   ⁵ Energy - Drinks
   ⁶ Cocoa
   ⁷ Other ________________
   ⁹ I don't want to answer that______

10. Do you bring drinks from home less often since the drinking fountain was introduced at your school?

    ¹ Yes ◎² No ◎⁹ Would not like to answer

11. Do you usually buy drinks during school hours? (For example, at the school kiosk or supermarket?)

    ¹ Yes ◎² No ◎⁹ Would not like to answer

12. If so, what drinks do you buy?
    ¹ Water
    ² Juice spritzers
    ³ Lemonades
    ⁴ Caffeinated soft drinks
    ⁵ Energy - Drinks
    ⁶ Cocoa
    ⁷ Other ________________
    ⁹ I don't want to answer that

We would also like to know your opinion on the following statements:

		Totally agree[1]	Partly true[2]	Rather not true[3]	Not true at all[4]	I do not want to answer[9]
13.	Drinking fountains at our school are a good idea	◎	◎	◎	◎	◎
14.	Other schools should also get drinking fountains	◎	◎	◎	◎	◎
15.	I drink more water since the drinking fountain has been there	◎	◎	◎	◎	◎
16.	Other children/young people from my class/school use the drinking fountain regularly.	◎	◎	◎	◎	◎
17.	When I choose a drink, I make sure it is healthy	◎	◎	◎	◎	◎

19. Who are you most likely to ask if you have questions about healthy drinks?

[1] My parents
[2] My teachers
[3] My friends
[4] I look for the information on the internet
[9] I don't want to answer that

		Totally agree[1]	Partly true[2]	Rather not true[3]	Not true at all[4]	I do not want to answer[9]
19.	I can distinguish healthy from unhealthy drinks	◎	◎	◎	◎	◎
20.	Healthy drinks were covered in class	◎	◎	◎	◎	◎

How healthy do you think the following drinks are?
(tick only one option at a time)

		Healthy[1]	Rather healthy[2]	Rather not healthy[3]	unhealthy[4]
21.	Water	◎	◎	◎	◎
22.	Juice spritzer	◎	◎	◎	◎

23.	Lemonades	◎	◎	◎	◎
24.	Caffeinated soft drinks	◎	◎	◎	◎
25.	Energy drinks	◎	◎	◎	◎
26.	Cocoa	◎	◎	◎	◎

Free text questions

27. Is there anything you would like to change about the drinking fountains? (For example, how they look or how they work)

____

____

28. If you would like to share something else with us on the subject of drinking fountains, you still have room here:

____

____

THANK YOU FOR YOUR PARTICIPATION!

Questionnaire S2. Teacher questionnaire containing 17 closed and two open items (German and English)

---

*Fragebogen für Lehrer*innen*

Nutzung und Akzeptanz von Trinkbrunnen
an Dortmunder Schulen

Sehr geehrte Lehrer*innen,
herzlichen Dank, dass auch Sie sich bereit erklärt haben, uns bei der wissenschaftlichen Auswertung des Trinkbrunnenprojekts Ihrer Schule zu unterstützen. Die nachfolgenden Fragen orientieren sich an dem Fragebogen, der auch den Schüler*innen ausgeteilt wurde. Es findet eine vollständige Anonymisierung der Daten statt, ein Rückschluss auf die Identität der ausfüllenden Person ist im Nachhinein nicht mehr möglich.

1. Geschlecht

   ◎1 Weiblich2 Männlich ◎3 Divers ◎9 Möchte ich nicht beantworten

______

2. Ihr Alter

   Ich bin __________ Jahre alt ◎9 Möchte ich nicht beantworten

______

3. An welcher Schulform sind Sie aktuell tätig?

   ◎1 Hauptschule ◎2 Gymnasium ◎9 Möchte ich nicht beantworten

______

4. Nutzen Sie den Trinkbrunnen in der Schule persönlich?

   ◎1 Ja ◎ 2 Nein ◎9 Möchte ich nicht beantworten

______

5. Wenn ja, wie oft haben Sie in der letzten Woche den Trinkbrunnen persönlich genutzt?

   ◎1 Mehr als 3-mal pro Tag
   ◎2 1 – 2-mal am Tag
   ◎3 Einmal am Tag
   ◎4 1-2-mal die Woche
   ◎5 Seltener als einmal die Woche
   ◎9 Möchte ich nicht beantworten

______

6. Gab es an Schulen, an denen Sie früher tätig waren, Trinkbrunnen?

   ◎1 Ja ◎ 2 Nein ◎9 Möchte ich nicht beantworten

7. Bringen Sie normalerweise Getränke von zu Hause mit zur Arbeit?

◎[1] Ja ◎[2] Nein ◎[9] Möchte ich nicht beantworten

8. Wenn ja, welche Getränke bringen Sie regelhaft von zu Hause mit? (Mehrfachantworten möglich)

◎[1] Wasser
◎[2] Saftschorlen
◎[3] Limonaden
◎[4] Koffeinhaltige Limonaden
◎[5] Energy – Drinks
◎[6] Kakao
◎[7] Andere ________________
◎[9] Möchte ich nicht beantworten

9. Bringen Sie seltener Getränke von zu Hause mit, seitdem es den Trinkbrunnen an der Schule gibt?

◎[1] Ja ◎[2] Nein ◎[9] Möchte ich nicht beantworten

Außerdem interessiert uns Ihre Meinung zu folgenden Aussagen

		Stimmt voll und ganz[1]	Stimmt teilweise[2]	Stimmt eher nicht[3]	Stimmt gar nicht[4]	Möchte ich nicht beantworten[9]
10.	Trinkbrunnen an unserer Schule sind eine gute Idee	◎	◎	◎	◎	◎
11.	Andere Schulen sollten auch Trinkbrunnen bekommen	◎	◎	◎	◎	◎
12.	Ich trinke mehr Wasser, seitdem es den Trinkbrunnen gibt	◎	◎	◎	◎	◎
13.	Mein Trinkverhalten wird durch meine Kollegen beeinflusst.	◎	◎	◎	◎	◎
14.	Wenn ich mir ein Getränk auswähle, achte ich darauf, dass es gesund ist	◎	◎	◎	◎	◎

15. Ich informiere mich über gesunde Getränke am ehesten …

◎[1] Durch Gespräche mit Kollegen oder Freunden
◎[2] Durch Gespräche mit Fachpersonen (bspw. Mediziner*innen)

◎3 Durch Printmedien
◎4 Durch Internetrecherche
◎9 Möchte ich nicht beantworten

		Stimmt voll und ganz[1]	Stimmt teilweise[2]	Stimmt eher nicht[3]	Stimmt gar nicht[4]	Möchte ich nicht beantworten[9]
16.	Ich habe das Thema gesunde Getränke in meinem Unterricht behandelt	◎	◎	◎	◎	◎
17.	Wenn ja, haben die Schüler*innen Nachfragen zum Thema gesunde Getränke gestellt?	◎	◎	◎	◎	◎

Freitextfragen

18. Gibt es etwas, das Sie am Trinkbrunnen gerne ändern würden?
(bspw. Aussehen, Funktion etc.)

____

19. Wenn Sie uns darüber hinaus noch etwas in Bezug auf den Trinkbrunnen mitteilen möchten, haben Sie hier Platz dafür:

____

VIELEN DANK FÜR IHRE TEILNAHME!

Questionnaire for teachers

Use and acceptance of drinking fountains
at Dortmund schools

Dear teachers,
Thank you very much for agreeing to support us in the scientific evaluation of your school's drinking fountain project. The following questions are based on the questionnaire that was also handed out to the pupils. The data will be completely anonymised and it will not be possible to identify the person who filled in the questionnaire.

13. Gender

[1] Female[2] Male ◎[3] Divers ◎[9] Would not like to answer

______

14. Your age

I am __________ years old ◎[9] I do not want to answer this question

______

15. At which type of school are you currently working?

[1] Hauptschule ◎[2] Gymnasium ◎[9] I do not wish to answer this question.

______

16. Do you personally use the drinking fountain at school?

[1] Yes ◎[2] No ◎[9] Would not like to answer

______

17. If yes, how often have you personally used the drinking fountain in the last week?

[1] More than 3 times a day
[2] 1 - 2 times a day
[3] Once a day
[4] 1-2 times a week
[5] Less often than once a week
[9] I don't want to answer that

______

18. Were there drinking fountains at schools where you used to work?

[1] Yes ◎[2] No ◎[9] Would not like to answer

______

19. Do you usually bring drinks from home to work?

[1] Yes ◎[2] No ◎[9] Would not like to answer

______

20. If yes, which drinks do you regularly bring from home? (Multiple answers possible)

[1] Water
[2] Juice spritzers
[3] Lemonades
[4] Caffeinated soft drinks
[5] Energy - Drinks
[6] Cocoa
[7] Other ________________
[9] I don't want to answer that

______

21. Do you bring drinks from home less often since the drinking fountain was installed at the school?

[1] Yes ◎[2] No ◎[9] Would not like to answer

We are also interested in your opinion on the following statements

---

		Totally agree[1]	Partly true[2]	Rather not true[3]	Not true at all[4]	I do not want to answer[9]
10.	Drinking fountains at our school are a good idea	◎	◎	◎	◎	◎
11.	Other schools should also get drinking fountains	◎	◎	◎	◎	◎
12.	I drink more water since the drinking fountain has been there	◎	◎	◎	◎	◎
13.	My drinking behaviour is influenced by my colleagues.	◎	◎	◎	◎	◎
14.	When I choose a drink, I make sure it is healthy	◎	◎	◎	◎	◎

16. I am most likely to inform myself about healthy drinks ...

[1] Through conversations with colleagues or friends
[2] Through conversations with professionals (e.g. doctors)
[3] Through print media
[4] Through internet research
[9] I don't want to answer that

		Totally agree[1]	Partly true[2]	Rather not true[3]	Not true at all[4]	I do not want to answer[9]
19.	I have covered the topic of healthy drinks in my lessons	◎	◎	◎	◎	◎
20.	If so, did the students ask questions about healthy drinks?	◎	◎	◎	◎	◎

Free text questions

---

21. Is there anything you would like to change about the drinking fountain?
    (e.g. appearance, function etc.)

____

22. If you would like to tell us anything else about the drinking fountain, you can do so here:

____

THANK YOU FOR YOUR PARTICIPATION!

Topic guide S1. Topic guide for a short interview of two school principals (German and English)

Leitfaden- Fragen für Interviews für Schulleiter*innen

1. Was musste getan werden, um das Trinkbrunnenprojekt zu realisieren?
    - (Prompts: Finanzierung, Genehmigungen, Dauer, ein oder verschiedene Ansprechpartner in der Stadt?)
2. Was ist Ihr Resümee bezüglich des Trinkbrunnenprojekts an Ihrer Schule?
3. Welche Empfehlungen würden Sie anderen Schulleiter*innen geben, die Interesse an einem Trinkbrunnen an ihrer Schule haben?

Guiding questions for interviews for head teachers

1. What had to be done to realise the drinking fountain project?
    - (Prompts: funding, permits, duration, one or different contacts in the city?)
2. What is your summary of the drinking fountain project at your school?
3. What recommendations would you give to other head teachers who are interested in having a drinking fountain at their school?

Figure S1. Comparison of students' responses from the two schools to selected questions about using and accepting drinking fountains

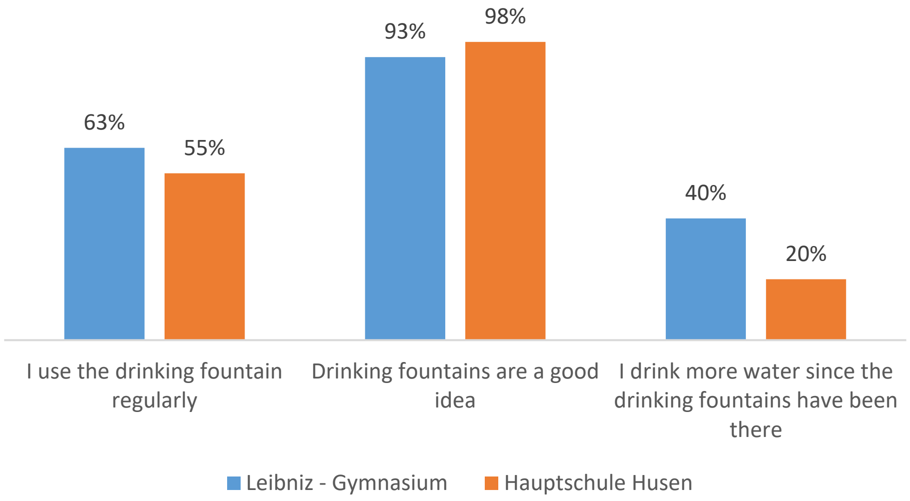

Table S1. Univariate ordinal logistic regression for risk factors of students' use of drinking fountains using students' responses to the statement "I use the drinking fountain regularly"

	Regression-Coefficient	Odds ratio	Lower 95% CI	Upper 95% CI
Gender	0,491	1,635	0,901	2,964
Age	0,000	1,000	0,998	1,002
School type	-0,006	0,994	0,947	1,044
Year	0,000	1,000	0,997	1,004
Water dispenser in the primary school	0,008	1,008	0,968	1,050
Drinks from home	-0,346	0,707	0,258	1,938
Peer Group	0,001	1,001	0,999	1,003
Lessons healthy drinks	-0,001	0,999	0,998	1,001

Table S2. Univariate ordinal logistic regression for risk factors of students' attitudes towards drinking fountains using students’ responses to the statement "drinking fountains are a good idea"

	**Regression-Coefficient**	**Odds ratio**	**Lower 95% CI**	**Upper 95% CI**
Gender	0,356	1,428	0,770	2,646
Age	7E-5	1	0,998	1,002
School type	-0,026	0,975	0,625	1,519
Year	0,001	1,001	0,998	1,004
Water dispenser in the primary school	0,001	1,001	1,000	1,003
Drinks from home	-0,012	0,988	0,690	1,414
Peer Group	0,000	1,000	0,999	1,002
Lessons healthy drinks	0,000	1,000	0,999	1,002

Table S3: Teachers' responses to the 17 closed questions in the teacher questionnaire (n=10)

	Quantity	%
1 Gender		
Female	6	60
Male	4	40
3. School type*		
Hauptschule	4	40
Gymnasium	6	60
4. Do you use the drinking fountain at school?		
Yes	4	40
no	6	60
5. How often do you use the drinking fountain?		
Less frequently than once a week	2	20
Would not like to answer	2	20
No answer given	6	60
6. Were there drinking fountains at schools where you used to work?		
Yes	1	10
No	8	80
Would not like to answer	1	10
7. Do you usually bring drinks from home to work?		
Yes	8	80
No	2	20
8. If yes, what drinks do you regularly bring from home? (multiple answers possible)		
Water	7	63,6
Coffee	2	18,2
Tea	2	18,2
9. Do you bring drinks from home less often since the drinking fountain is at the school?		
Yes	3	30
No	7	70
10. Drinking fountains at our school are a good idea		
Fully agrees	8	80
Partly true	2	20
11. Other schools should also get drinking fountains		
Fully agrees	7	70
Partly true	2	20
Would not like to answer	1	10
12. I drink more water since the drinking fountain has been available		
Fully agrees	1	10
Partly true	1	10
Rather not true	3	30
Not true at all	5	50
13. My colleagues influence my drinking behaviour		
Rather not true	4	40
Not true at all	6	60
14. When I choose a drink, I make sure that it is healthy		
Fully agrees	6	60
Partly true	4	40
15. I am most likely to inform myself about healthy drinks (multiple		

answers allowed)		
Through conversations with colleagues or friends	1	9
Through discussions with specialists (e.g. physicians)	1	9
Through print media	1	9
Through internet research	5	45,5
Would not like to answer	3	27,3
16. I have covered the topic of healthy drinks in my classes		
Fully agrees	5	50
Partly true	1	10
Rather not true	1	10
Not true at all	3	30
17. If so, did the students ask follow-up questions about the topic of healthy drinks provided?		
Fully agrees	2	28,6
Partly true	2	28,6
Not true at all	3	42,9

*The table does not list the results from question 2 about the teachers' age
